# Platelet-to-Lymphocyte and Glucose-to-Lymphocyte Ratios as Prognostic Markers in Hospitalized Patients with Acute Coronary Syndrome

**DOI:** 10.3390/jcdd12090334

**Published:** 2025-08-30

**Authors:** Christos Kofos, Andreas S. Papazoglou, Barbara Fyntanidou, Athanasios Samaras, Panagiotis Stachteas, Athina Nasoufidou, Aikaterini Apostolopoulou, Paschalis Karakasis, Alexandra Arvanitaki, Marios G. Bantidos, Dimitrios V. Moysidis, Nikolaos Stalikas, Dimitrios Patoulias, Apostolos Tzikas, George Kassimis, Nikolaos Fragakis, Efstratios Karagiannidis

**Affiliations:** 1 Second Department of Cardiology, General Hospital ‘Hippokration’, Aristotle University of Thessaloniki, Konstantinoupoleos 49, 54642 Thessaloniki, Greece; chriskofos21@gmail.com (C.K.); asamaraa@auth.gr (A.S.); athinaso@auth.gr (A.N.); pkarakaa@auth.gr (P.K.); aarvanic@med.auth.gr (A.A.); aptzikas@yahoo.com (A.T.); gkasimis@auth.gr (G.K.); nfrag@auth.gr (N.F.); 2 Department of Cardiology, Athens Naval Hospital, 11521 Athens, Greece; 3 Department of Emergency Medicine, AHEPA University Hospital, 54636 Thessaloniki, Greece; fyntanidou@auth.gr (B.F.); aapostoo@auth.gr (A.A.); mpantidos@auth.gr (M.G.B.); 4 Medical School, Aristotle University of Thessaloniki, 54124 Thessaloniki, Greece; dimoysidis@gmail.com; 5 Cardiovascular Center, AZORG Ziekenhuis, Moorselbaan 164, 9300 Aalst, Belgium; nikolaos.stalikas@azorg.be; 6 Second Propaedeutic Department of Internal Medicine, Faculty of Medicine, School of Health Sciences Aristotle, University of Thessaloniki, 54124 Thessaloniki, Greece; patoulias@auth.gr

**Keywords:** acute coronary syndrome, platelet-to-lymphocyte ratio, PLR, glucose-to-lymphocyte ratio, GLR, inflammatory biomarkers, all-cause mortality, prognostic ratios

## Abstract

Background: Novel and accessible biomarkers may add to the existing risk stratification schemes in patients with acute coronary syndrome (ACS). The platelet-to-lymphocyte ratio (PLR) and glucose-to-lymphocyte ratio (GLR) have emerged as potential indicators of systemic inflammation and metabolic stress, both of which are pivotal in ACS pathophysiology. The aim of this study was to investigate the prognostic significance of the PLR and GLR in patients with ACS. Methods: We performed a retrospective cohort study of patients hospitalized with ACS between 2017 and 2023 at Hippokration Hospital of Thessaloniki, Greece. PLR and GLR were calculated from admission blood samples. The primary endpoint was all-cause mortality. Logistic and Cox regression models were used to investigate the associations of PLR and GLR with all-cause mortality. Receiver operating characteristic (ROC) analysis, Kaplan–Meier survival curves, and restricted cubic spline (RCS) modeling were also applied. Results: In total, 853 patients (median age: 65 years, 72.3% males) were included. Higher PLR and GLR were independently associated with increased risk of long-term mortality [adjusted Odds Ratio (aOR) for PLR: 1.007, 95% CI: 1.005–1.008; and for GLR: aOR = 1.006, 95% CI: 1.003–1.008]. The optimal cut-off values were 191.92 for PLR and 66.80 for GLR. Kaplan–Meier and Cox regression analyses confirmed significantly reduced survival in patients with GLR and PLR values exceeding these thresholds. RCS analysis revealed non-linear relationships, with mortality risk rising sharply at higher levels of both markers. PLR showed superior prognostic performance (AUC: 0.673, 95% CI: 0.614–0.723) compared to GLR (AUC: 0.602, 95% CI: 0.551–0.653). Conclusions: While PLR demonstrated greater predictive accuracy, both PLR and GLR were consistently associated with mortality and may provide complementary prognostic information. Incorporating those ratios into routine clinical assessment may improve risk stratification, particularly in resource-limited settings or for patients without traditional risk factors.

## 1. Introduction

Cardiovascular disease (CVD) remains the leading cause of mortality worldwide, with acute coronary syndrome (ACS) representing a significant contributor to morbidity and healthcare burden [1,2]. Despite advancements in early diagnosis and treatment, ACS is still associated with high short- and long-term mortality rates. Effective risk stratification is crucial for optimizing patient management, informing therapeutic decisions, and enhancing outcomes. Traditional risk assessment tools, such as the GRACE [3] and TIMI scores [4], provide valuable prognostic insights, but the need for additional, easily accessible biomarkers to refine risk prediction remains.

In recent years, inflammatory markers have gained attention as potential prognostic indicators in ACS, reflecting the critical role of systemic inflammation in CVD pathophysiology. Markers such as neutrophil-to-lymphocyte ratio (NLR), C-reactive protein (CRP), and interleukin-6 (IL-6) have shown independent associations with cardiovascular events, but their application in routine clinical settings is limited by factors such as cost, availability, and inconsistent standardization across laboratories [5,6,7].

The platelet-to-lymphocyte ratio (PLR) and glucose-to-lymphocyte ratio (GLR) have emerged as novel biomarkers with potential utility in patients’ prognostication. PLR reflects platelet activation and immune response [8], both of which contribute to atherosclerosis and thrombotic complications, while GLR integrates metabolic and inflammatory stress [9], which are strongly linked to cardiovascular risk. These ratios build on the same foundation as NLR, offering a composite view of inflammation, immune status, and, especially for GLR, metabolic stress.

Although both markers derive from routine blood tests, their clinical relevance in ACS prognosis remains under investigation, with limited knowledge on their optimal cut-off values and predictive power. Importantly, PLR and GLR are inexpensive, universally available, and rapidly obtainable, making them attractive for use in emergency and resource-limited settings, especially when other prognostic markers such as BNP or serial troponin testing are unavailable or delayed [10]. Blood-derived ratios such as NLR have been studied since the early 2010s in both oncology and cardiology as cost-effective inflammatory markers with prognostic potential [11,12]. These ratio-based indices may represent a practical extension of biomarker-based risk assessment.

This study aims to assess the prognostic value of PLR and GLR in predicting long-term mortality in ACS patients, potentially providing a cost-effective and widely available alternative for risk stratification.

## 2. Methods

### 2.1. Study Sample and Ethics

This retrospective cohort study analyzed consecutive patients hospitalized for ACS in the Second Department of Cardiology at Hippokration Hospital of Thessaloniki between 2017 and 2023. ACS diagnosis was established according to the Fourth Universal Definition of Myocardial Infarction (2018), incorporating clinical presentation, ECG changes, and cardiac biomarker elevation [13]. Ethical approval for this study was obtained from the Ethics Committee of Hippokration Hospital of Thessaloniki. The study was conducted following the principles outlined in the Declaration of Helsinki [14]. As this represents a retrospective study, individual informed consent was not required.

### 2.2. Definitions and Study Endpoint

PLR and GLR were calculated from blood samples obtained upon hospital admission, prior to any coronary intervention. High-sensitivity cardiac Troponin T (hs-TnT) was measured using the Roche Elecsys assay. The upper reference limit (99th percentile) for this assay was 14 ng/L. PLR was calculated as platelet count (×10^9^/L) divided by the absolute lymphocyte count (×10^9^/L), and GLR was calculated as glucose level (mg/dL) divided by the estimated absolute lymphocyte count (×10^9^/L). These ratios were selected over their components as they integrate multiple dimensions of risk into a single variable, such as inflammation, thrombosis, and metabolic stress, which are highly relevant to ACS pathophysiology and may provide more clinically interpretable insights than isolated markers.

The primary endpoint of this study was all-cause mortality during the follow-up period of approximately 2 years. All-cause mortality was defined as death due to any cause occurring after hospital admission.

### 2.3. Follow-Up Procedures

Patient follow-up was conducted through a combination of electronic health record review, telephone interviews, and, where necessary, contact with primary care physicians or family members to verify survival status. Mortality data were cross-checked against hospital records and national death registries to ensure accuracy.

### 2.4. Statistical Analysis

The normality of continuous variables was tested using the Shapiro–Wilk test [15]. Since most variables were non-normally distributed, medians and interquartile ranges (IQRs) are reported, while categorical variables are presented as frequencies and percentages. To evaluate whether PLR and GLR levels differed across ACS subtypes, we compared their values among patients with STEMI, NSTEMI, and unstable angina through Analysis of Variance (ANOVA) test. Associations between PLR, GLR, and mortality were assessed using univariate and multivariate logistic regression models, adjusting for clinically relevant covariates, including heart failure, gender, hypertension, diabetes mellitus, dyslipidemia, age, serum creatinine values, admission high-sensitivity troponin levels, and the use of medications such as anticoagulants (including direct oral anticoagulants (DOACs), low-molecular-weight heparin (LMWH), and unfractionated heparin (UFH), β-blockers, and diuretics as reported at admission. Receiver operating characteristic (ROC) curve analysis was performed to evaluate the predictive accuracy of PLR and GLR, with area under the curve (AUC) values reported and optimal cut-off values determined using Youden’s Index. In addition to analyzing PLR and GLR, we conducted univariate ROC curve analyses for their individual components, glucose, platelet count, and lymphocyte count, to assess their standalone predictive ability.

Cox proportional hazard regression analyses were conducted separately for PLR and GLR to assess their independent associations with mortality over time. Hazard ratios (HRs) with 95% confidence intervals (CIs) were estimated. Kaplan–Meier survival analysis [16] was used to compare survival distributions, stratifying patients by the optimal cut-off values of PLR and GLR, with comparisons made using the log-rank test. To improve interpretability and reduce the influence of extreme follow-up values, survival curves were truncated at the 90th percentile of follow-up duration. To further assess categorical risk, PLR and GLR were stratified into tertiles based on their distribution. Cox proportional hazards models were used to estimate hazard ratios for the middle and highest tertiles, using the lowest tertile as the reference.

To explore potential non-linear associations between GLR, PLR, and mortality, we applied restricted cubic spline (RCS) regression analyses [17] within a Cox proportional hazards model. Using four knots selected based on statistical criteria, we assessed how mortality risk varied across different values of GLR and PLR while adjusting for relevant clinical factors. This approach allowed us to capture complex patterns that a linear model might overlook, and the results were visualized using spline curves. In the resulting spline curves, the solid line represents the estimated HR across the range of the biomarker, while the shaded area reflects the 95% CIs. Lower CIs above 1 indicate significantly increased mortality risk, whereas upper CIs below 1 suggest significantly decreased mortality risk.

Statistical significance was set at *p* < 0.05, and all analyses were conducted using IBM SPSS Statistics (version 28.0) and R (version 4.4.2).

## 3. Results

A total of 853 patients were included, with a median age of 65 years (IQR: 56–75 years), and 72.3% were male. The median follow-up period was 25 months (IQR: 24–26 months). Among the patients included in the study, 36.4% presented with STEMI, 29.3% with NSTEMI, and 33.7% with unstable angina. The median PLR was 125.1 (IQR: 91.1–174.6), and the median GLR was 60.9 (IQR: 23.7–91.7). Serum creatinine had a median value of 0.98 mg/dL (IQR: 0.82–1.2 mg/dL). The most common comorbidities in the study population were arterial hypertension (49.2%), diabetes mellitus (27.2%), dyslipidemia (24.7%), and atrial fibrillation (8.6%), while 3.5% of patients had a history of heart failure (Table 1). Patients with increased PLR (≥191.92) were significantly older (median 67.5 vs. 63.6 years, *p* < 0.001), more likely to have diabetes mellitus (34.3% vs. 24.2%, *p* = 0.006), and had higher serum creatinine levels (1.56 vs. 1.11 mg/dL, *p* < 0.001) compared to those with lower PLR. Similarly, individuals with increased GLR (≥66.8) were also older (median 68 vs. 61 years, *p* = 0.003), had a higher prevalence of diabetes mellitus (40.0% vs. 15.7%, *p* < 0.001), and more frequently had hypertension (54.8% vs. 45.1%, *p* = 0.004), atrial fibrillation (13.7% vs. 5.4%, *p* < 0.001), and elevated serum creatinine levels (1.06 vs. 0.93 mg/dL, *p* = 0.004). PLR and GLR were compared across ACS subtypes (STEMI, NSTEMI, and unstable angina). PLR values differed significantly among groups (*p* = 0.036). Median PLR was highest in NSTEMI (132.6, IQR 99.6–185.3), followed by STEMI patients (126.8, IQR 90.4–174.0), and lowest in unstable angina (112.5, IQR 88.8–160.0). Post hoc pairwise comparisons with Bonferroni correction revealed a significant difference only between NSTEMI and unstable angina (*p* = 0.0047). GLR values followed a similar pattern, with medians of 68.1 (IQR 46.9–106.5) in STEMI, 59.9 (IQR 43.2–103.4) in NSTEMI, and 60.8 (IQR 43.8–88.5) in unstable angina; however, these differences did not reach statistical significance (*p* = 0.101).

**Figure 1:**
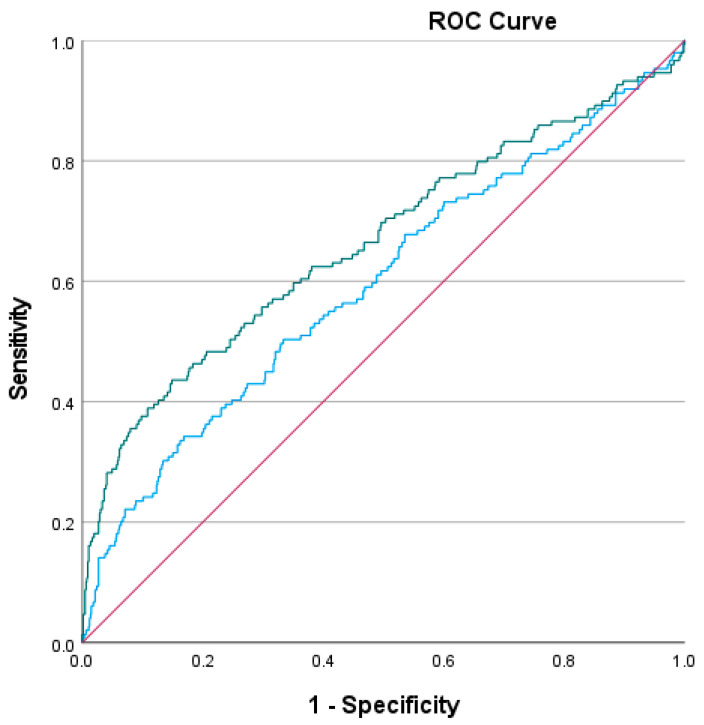
ROC curve for GLR (green line) and PLR (blue line).

Higher PLR (aOR: 1.006, 95% CI: 1.004–1.008, *p* < 0.001) and higher GLR (aOR: 1.005, 95% CI: 1.003–1.008, *p* < 0.001) were both independently associated with mortality. Other independent predictors of mortality included, dyslipidemia and age for the PLR-model, and dyslipidemia, anticoagulant use, diuretic use, and age for the GLR-model. (*p* < 0.05) (Supplementary Tables S1 and S2). In the ROC analysis, PLR demonstrated an AUC of 0.673 (95% CI: 0.614–0.723), with an optimal cutoff value of 191.92, while GLR had an AUC of 0.602 (95% CI: 0.551–0.653) (Figure 1), with an optimal cutoff of 66.80, using Youden’s Index. Using the DeLong test [18], we found that the AUC of the PLR model was significantly higher than that of the GLR model (*p* = 0.026). In univariate ROC analysis, individual components of the PLR and GLR were evaluated; however, they showed modest-to-low discriminatory ability. Glucose yielded an AUC of 0.578 (95% CI: 0.527–0.629), platelet count an AUC of 0.532 (95% CI: 0.482–0.582), and lymphocyte count an AUC of 0.366 (95% CI: 0.314–0.417).

**Figure 2:**
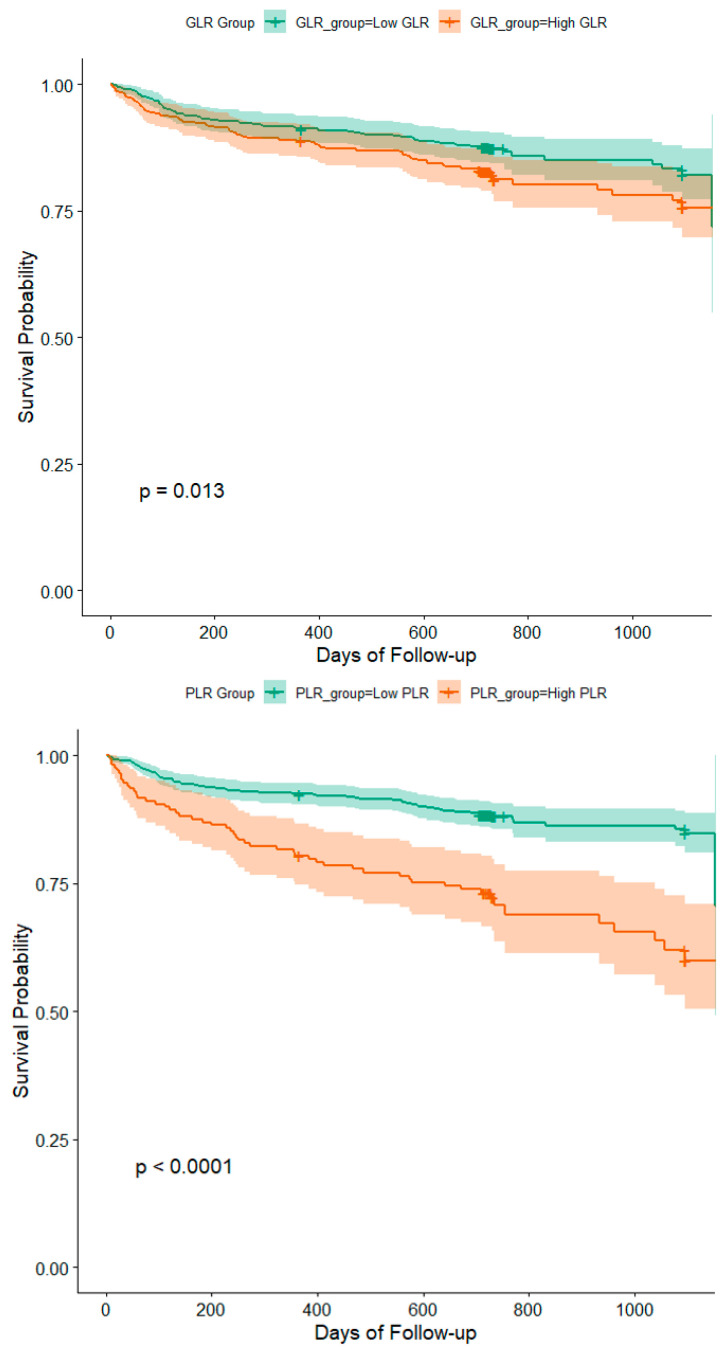
Kaplan–Meier survival plots for GLR at the top (green line: low GLR, orange line: high GLR) and for PLR at the bottom (green line: low PLR, orange line: high PLR) truncated at 1096 days (90th percentile of follow-up). Shaded areas represent 95% confidence intervals.

Multivariate Cox regression analysis confirmed that PLR was an independent predictor of mortality over time (aHR: 1.002, 95% CI: 1.001–1.002, *p* < 0.001), as well as GLR was also independently associated with mortality (aHR: 1.002, 95% CI: 1.001–1.003, *p* = 0.004) (Supplementary Tables S3 and S4). In all supplementary regression analyses (Supplementary Tables S1–S4), GLR and PLR were treated as continuous variables. Both markers remained independently associated with mortality after multivariable adjustment. Kaplan–Meier survival analysis was performed using dichotomized GLR and PLR values, with thresholds determined by the Youden Index from ROC analysis (66.8 for GLR and 191.92 for PLR). To improve clarity and avoid overextension due to a few patients with long follow-up durations, curves were truncated at 1096 days, corresponding to the 90th percentile of observed follow-up. In the top panel of Figure 2, patients with GLR above 66.8 exhibited significantly reduced survival compared to those with lower GLR (*p* = 0.013). Similarly, in the bottom panel, patients with PLR above 191.92 had markedly worse survival (*p* < 0.0001). Group separation became more distinct beyond the first year in both cases, and statistical significance was confirmed using log-rank testing. PLR tertiles were defined as: low (≤103.0), middle (103.1–153.0), and high (>153.0). GLR tertiles were defined as: low (≤48.6), middle (48.7–79.4), and high (>79.4). When analyzed by tertiles, both PLR and GLR showed significant prognostic stratification. Patients in the highest PLR tertile had more than double the risk of mortality compared to those in the lowest tertile (aHR: 2.041, 95% CI: 1.353–3.082, *p* < 0.001), while no significant difference was observed for the middle tertile (aHR: 1.102, 95% CI: 0.693–1.751, *p* = 0.690). Similarly, patients in the highest GLR tertile had a significantly elevated mortality risk (aHR: 2.261, 95% CI: 1.471–3.483, *p* < 0.001), with no significant difference for the middle tertile (aHR: 1.114, 95% CI: 0.682–1.811, *p* = 0.673).

**Figure 3:**
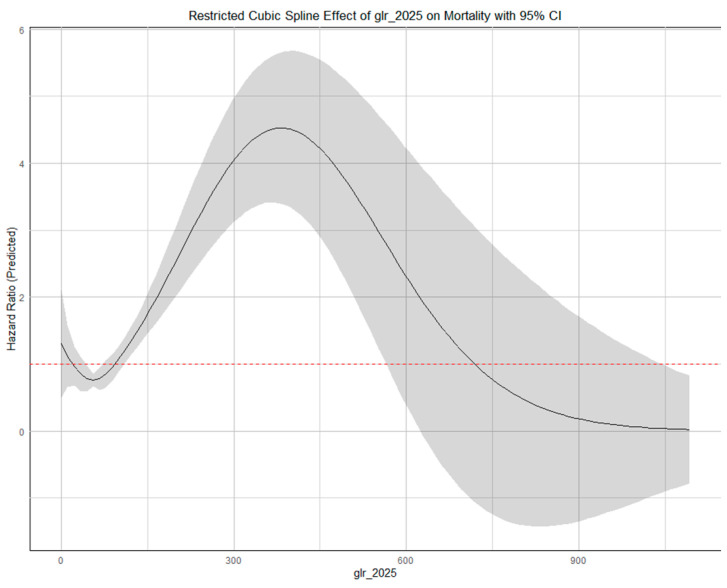
Restricted Cubic Spline for glucose-to-lymphocyte ratio.

**Figure 4:**
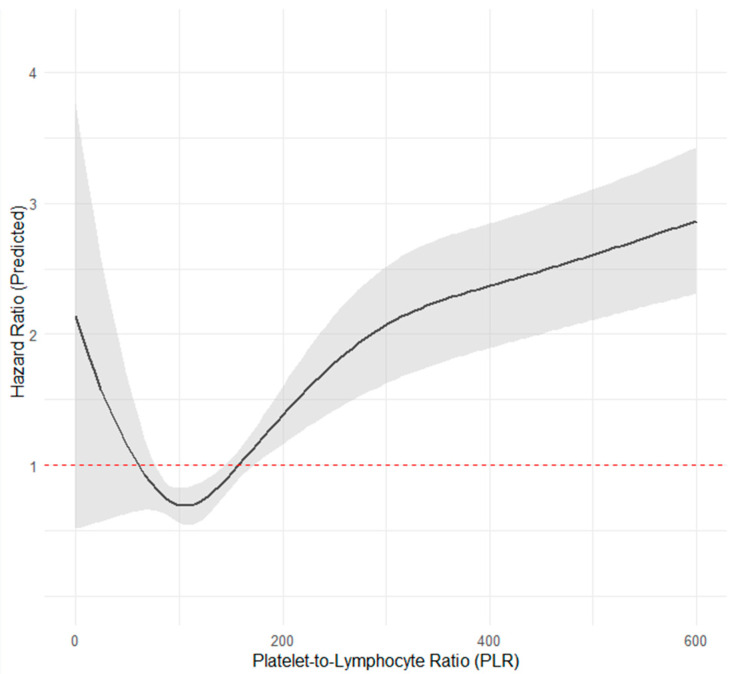
Restricted Cubic Spline for platelet-to-lymphocyte ratio.

The RCS analysis showed that the relationship between GLR, PLR, and mortality was not strictly linear. For GLR, the model identified a significant increase in mortality risk at the range of GLR values between 120 and 570 (Figure 3), while the remaining GLR levels had non-significant associations. Although the GLR spline curve appears to flatten in its upper range (GLR > 750), this segment represents a small number of patients (upper 1.05% percentile of the GLR values), where the wide 95% confidence intervals reflect limited statistical precision. Nevertheless, the highest GLR tertile, defined as >79.4, captures the portion of the curve where the risk is most clearly elevated. The RCS plot on PLR demonstrated a non-linear J-shaped trend, with a significant increase in mortality risk when PLR was above 174 (Figure 4).

## 4. Discussion

This study aimed to assess the prognostic value of PLR and GLR as markers for long-term mortality in patients with ACS. Our findings showed that both PLR and GLR were independent predictors of mortality, with higher values associated with worse outcomes after adjusting for traditional risk factors. While both PLR and GLR were independently associated with mortality, their discriminative ability was only modest, as reflected by their AUC values. Notably, PLR outperformed GLR in predictive accuracy, suggesting that it may be a more reliable prognostic biomarker in this clinical context. In tertile-based models, both PLR and GLR showed a significant stepwise association with long-term mortality. Specifically, the highest tertiles of both markers were associated with a twofold increased risk of death, supporting their utility as stratification tools. These findings align with spline model results and reinforce the role of elevated PLR and GLR as independent prognostic indicators in ACS.

It is important to note that these ratios integrate multiple biological processes, such as inflammation, immune suppression, and metabolic stress, into a single marker. This allows them to reflect complex risk states more meaningfully than their individual components, such as glucose or lymphocyte count alone. Also, we need to consider that despite their lower AUCs compared to established biomarkers such as troponin or BNP, PLR and GLR offer independent prognostic value and may serve as accessible, complementary tools, particularly in settings where serial cardiac biomarker testing is limited or unavailable.

Our results support those of several recent studies highlighting the value of PLR and GLR as prognostic biomarkers. The association between elevated PLR and adverse cardiovascular outcomes has been previously established. Platelet activation, as reflected by PLR, plays a crucial role in the thrombotic processes leading to atherosclerotic plaque rupture. Zhou et al. [19] highlighted that increased platelet activity is linked to more severe coronary lesions, reinforcing the observation that higher PLR values correlate with worse outcomes in our cohort. Wahyuni et al. [20] revealed a significant correlation between higher PLR and complex coronary lesions. This study demonstrated a highly predictive AUC of 93.3% in patients aged ≤ 45 years, with an optimal PLR cut-off of 111.06, while for those >45 years, the AUC was 77.3%. In contrast, our study demonstrated a moderate AUC of 67.3% for PLR (cut-off: 191.92) in predicting mortality risk, indicating a less precise but still significant role for PLR. In addition, Pruc et al. [21] found that a higher PLR was independently associated with an increased risk of major adverse cardiovascular events, including cardiovascular death, myocardial infarction, and stroke, in patients with ACS. In addition, Oylumlu et al. [22] highlighted the role of platelets in long-term mortality in ACS patients, with the highest tertile showing significantly worse outcomes over a 5-year follow-up.

Moreover, our study is among the few to explore the prognostic value of GLR in ACS. GLR integrates both glucose metabolism and immune response, reflecting the broader systemic inflammation and metabolic stress. According to recent research [23,24,25], hyperglycemia is linked to increased endothelial dysfunction, platelet aggregation, and oxidative stress, all of which are key contributors to ACS progression. Liu et al. [26], in their study on GLR as a prognostic factor for all-cause mortality in STEMI patients, found similar results, with increased GLR associated with poor outcomes. This study demonstrated a significant relationship between higher GLR levels and increased risk of in-hospital mortality, with an HR of 1.70 (95% CI: 1.24–2.34) after adjusting for several clinical covariates. Liu et al. also utilized RCS analysis and demonstrated a non-linear relationship between GLR and in-hospital mortality risk, with a cut-off GLR value of 104.66. This suggests that beyond this threshold, the risk of mortality rises significantly. In the study by Serhatlioglu et al. [27], GLR was a strong predictor of critical coronary artery disease (CAD), with an AUC of 0.801 (utilizing a cut-off value of 41.2), sensitivity of 83.9%, and specificity of 73%. This highlights the clinical utility of GLR in identifying high-risk patients with severe CAD. In comparison, our study shows that GLR remains an important prognostic marker in ACS patients, particularly for predicting long-term mortality. While the AUC of 0.602 observed in our analysis is somewhat lower, it still reinforces the potential of GLR as a valuable, cost-effective biomarker.

## 5. Limitations

Despite the strengths of this study, several limitations must be acknowledged. One of the main limitations is its retrospective design, which inherently carries the risk of selection bias. Additionally, the single center conduction of this study potentially restricts the generalizability of our findings to broader populations. The sample size, while adequate for statistical analysis, may still limit the robustness of our conclusions, particularly when providing the ratio cut-offs and when stratifying patients based on biomarker thresholds. Another limitation is the inability to define specific cut-off values for PLR and GLR, as the RCS analysis indicated non-linear continuous relationships with mortality. This suggests that thresholds derived with Youden’s Index may not fully capture the complexity of these associations. Future studies should consider incorporating RCS modeling to better reflect the continuous nature of these biomarkers and refine their clinical applicability. Furthermore, PLR and GLR are influenced by various physiological and pathological conditions, such as infections, malignancies, and other inflammatory diseases, which could not be sufficiently addressed in our multivariate analyses. Lastly, the two ratios were assessed only at patient admission; hence, no dynamic changes in inflammatory markers over time could be assessed, which may influence their predictive capacity. Due to retrospective design and inconsistent follow-up testing, serial changes in PLR and GLR during hospitalization could not be evaluated. Finally, we would like to emphasize that PLR and GLR are best viewed as complementary to, rather than as substitutes for, established prognostic biomarkers such as peak hs-TnT or admission glucose. These limitations underscore the need for future prospective, multicenter studies to validate our findings.

## 6. Conclusions

In conclusion, our study provides further evidence that both PLR and GLR are valuable biomarkers for predicting long-term mortality in ACS patients. While PLR demonstrated superior discriminatory power compared to GLR, both markers proved to be effective in survival prediction, highlighting the role of systemic inflammation in the prognosis of ACS. Given the simplicity and cost-effectiveness of PLR and GLR, their inclusion in clinical protocols could improve patient management, particularly in resource-limited settings where more complex tools are not readily accessible and also when dealing with patients without traditional risk factors where more personalized approaches are needed. Further research is needed to confirm these findings and explore how these biomarkers can be integrated into existing clinical decision-making frameworks for post-ACS risk stratification.

## Figures and Tables

**Table 1:** Baseline characteristics of the study population. Elevated PLR was defined as ≥191.92 (n = 178) and elevated GLR as ≥66.8 (n = 396). Elevated GLR and PLR were defined based on optimal cut-off values derived from ROC curve analysis using the Youden index. Values are presented as median (IQR) or percentage.

Variable	Total Population (N = 853)	Elevated PLR (≥191.92)	Not-Elevated PLR (<191.92)	*p*-Value (PLR)	Elevated GLR (≥66.8)	Not-Elevated GLR (<66.8)	*p*-Value (GLR)
**Age (years)**	65 (56–75)	67.50 (IQR 22)	63.55 (IQR 18)	<0.001	68 (IQR 19)	61 (IQR 18)	0.003
**Male sex (%)**	72.3%	17.7%	82.3%	0.003	68.5%	22.7%	0.003
**Diabetes Mellitus (%)**	27.2%	34.3%	24.2%	0.006	40.0%	15.7%	<0.001
**Hypertension (%)**	49.2%	49.7%	49.2%	0.893	54.8%	45.1%	0.004
**Dyslipidemia (%)**	24.7%	26.6%	25.4%	0.759	27.8%	24.0%	0.208
**Serum Creatinine (mg/dL), median (IQR)**	0.98 (0.82–1.2)	1.56 (IQR 0.63)	1.11 (IQR 0.32)	<0.001	1.06 (IQR 0.49)	0.93 (IQR 0.29)	0.004
**Statin use**	48.8	47.4	50.6	0.509	47.9	47.9	1.000
**Anticoagulant use**	20.6	17.5	27.0	0.006	15.6	24.5	0.001
**Β-blockers use**	49.2	45.1	58.0	0.003	46.3	49.5	0.392
**Heart Failure**	3.2	1.7	8.0	<0.001	1.5	4.8	0.010
**Chronic Kidney Disease**	7.4	5.2	15.3	<0.001	3.7	12.3	<0.001
**Smoking status**	43.7	48.4	34.3	0.001	52.3	36.8	<0.001
**HS-Troponin T**	116.00 (13.00–1182.00)	94.00 (11.50–1024.00)	385.15 (42.50–2258.00)	<0.001	93.00 (11.00–809.40)	256.00 (19.50–1836.00)	<0.001
**Atrial Fibrillation, n (%)**	8.6%	12.3%	8.2%	0.103	13.7%	5.4%	<0.001

Abbreviations: 1. **PLR** = Platelet-to-Lymphocyte Ratio; 2. **GLR** = Glucose-to-Lymphocyte Ratio; 3. **IQR** = Interquartile Range; 4. **mg/dL** = milligrams per deciliter.

## Data Availability

Study data will be available upon reasonable request from the corresponding study author (E.K.).

## Supplementary Materials

The following supporting information can be downloaded at: https://www.mdpi.com/article/10.3390/jcdd12090334/s1, Table S1: Univariate and Multivariable Logistic Regression Analysis for GLR as a continuous variable; Table S2: Univariate and Multivariable Logistic Regression Analysis for PLR as a continuous variable; Table S3: Univariate and Multivariable Cox Regression Analysis for GLR as a continuous variable; Table S4; Univariate and Multivariable Cox Regression Analysis for PLR as a continuous variable.
