# Supplementary material for: Platelet-to-Lymphocyte and Glucose-to-Lymphocyte Ratios as Prognostic Markers in Hospitalized Patients with Acute Coronary Syndrome
Source: J Cardiovasc Dev Dis. 2025 Aug 30;12(9):334. doi: 10.3390/jcdd12090334 (PMC12470989; doi:10.3390/jcdd12090334)
Supplement: Supplementary file 1 [file jcdd-12-00334-s001.zip › jcdd-3701182-supplementary.pdf]

Supplementary Table S1. Univariate and Multivariable Logistic regression analysis for GLR as a continuous variable.

**Variable**	**OR (95% CI)**	**p value**	**aOR (95% CI)**	**p value**
**GLR**	1.497 (1.224–1.830)	<0.001	1.005 (1.003–1.008)	<0.001
Heart Failure	4.038 (1.806–9.029)	<0.001	1.954 (0.696–5.482)	0.203
Gender	0.793 (0.552–1.140)	0.210	1.318 (0.815–2.133)	0.260
Arterial Hypertension	0.945 (0.676–1.320)	0.739	0.771 (0.488–1.218)	0.265
Diabetes Mellitus	2.119 (1.492–3.011)	<0.001	1.253 (0.773–2.032)	0.360
Dyslipidemia	0.613 (0.402–0.936)	0.024	0.553 (0.328–0.933)	0.026
Chronic Kidney Failure	3.868 (2.249–6.651)	<0.001	1.422 (0.631–3.203)	0.396
Atrial Fibrillation	2.083 (1.251–3.468)	0.005	0.770 (0.382–1.554)	0.466
Anticoagulant use	2.149 (1.473–3.135)	<0.001	1.733 (1.030–2.917)	0.038
Diuretic use	2.604 (1.800–3.768)	<0.001	1.666 (1.022–2.716)	0.039
B-blockers use	1.492 (1.064–2.092)	0.020	1.124 (0.714–1.772)	0.613
Age	1.048 (1.033–1.063)	<0.001	1.024 (1.006–1.044)	0.010
tnt_HS	1.000 (1.001–1.003)	0.047	1.000 (1.001–1.003)	0.943
Serum creatinine	1.506 (1.274–1.779)	<0.001	1.150 (0.942–1.405)	0.169

Supplementary Table S2: Univariate and Multivariable Logistic regression analysis for PLR as a continuous variable.

**Variable**	**OR (95% CI)**	**p value**	**aOR (95% CI)**	**p value**
**PLR**	1.497 (1.224–1.830)	<0.001	1.006 (1.004–1.008)	<0.001
Heart Failure	4.038 (1.806–9.029)	<0.001	2.222 (0.776–6.359)	0.137
Gender	0.793 (0.552–1.140)	0.210	1.485 (0.906–2.435)	0.117
Arterial Hypertension	0.945 (0.676–1.320)	0.739	0.729 (0.457–1.163)	0.185
Diabetes Mellitus	2.119 (1.492–3.011)	<0.001	1.514 (0.931–2.462)	0.094
Dyslipidemia	0.613 (0.402–0.936)	0.024	0.548 (0.322–0.933)	0.027
Chronic Kidney Failure	3.868 (2.249–6.651)	<0.001	1.093 (0.457–2.615)	0.842
Atrial Fibrillation	2.083 (1.251–3.468)	0.005	0.852 (0.416–1.744)	0.660
Anticoagulant use	2.149 (1.473–3.135)	<0.001	1.591 (0.939–2.696)	0.084
Diuretic use	2.604 (1.800–3.768)	<0.001	1.540 (0.931–2.549)	0.093
B-blockers use	1.492 (1.064–2.092)	0.020	0.988 (0.624–1.565)	0.959
Age	1.048 (1.033–1.063)	<0.001	1.025 (1.006–1.044)	0.008
tnt_HS	1.000 (1.001–1.003)	0.047	1.000 (1.001–1.003)	0.803
Serum creatinine	1.506 (1.274–1.779)	<0.001	1.214 (0.989–1.492)	0.064

Supplementary Table S3. Univariate and Multivariable Cox Regression Analysis for GLR as a continuous variable.

Variable	HR (95% CI)	p value	aHR (95% CI)	p value
GLR	1.002 (1.002–1.003)	<0.001	1.002 (1.001–1.003)	0.004
Heart Failure	3.822 (2.111–6.922)	<0.001	2.438 (1.190–4.993)	0.015
Gender	0.927 (0.670–1.284)	0.649	1.199 (0.798–1.800)	0.383
Arterial Hypertension	0.969 (0.716–1.311)	0.838	0.933 (0.631–1.379)	0.728
Diabetes Mellitus	1.629 (1.192–2.227)	0.002	1.171 (0.782–1.753)	0.444
Dyslipidemia	0.845 (0.568–1.259)	0.408	0.831 (0.524–1.317)	0.431
Chronic Kidney Failure	3.095 (2.040–4.695)	<0.001	1.231 (0.675–2.248)	0.498
Atrial Fibrillation	1.718 (1.118–2.641)	0.014	1.046 (0.594–1.843)	0.877
Anticoagulant use	1.565 (1.124–2.179)	0.008	1.177 (0.759–1.825)	0.467
Diuretic use	2.058 (1.493–2.835)	<0.001	1.712 (1.163–2.519)	0.006
B-blockers use	1.117 (0.819–1.524)	0.484	0.870 (0.593–1.277)	0.477
Age	1.031 (1.018–1.044)	<0.001	1.016 (1.000–1.032)	0.051
tnt_HS	1.001 (1.001–1.003)	0.004	1.001 (1.001–1.003)	0.286
Serum creatinine	1.202 (1.104–1.308)	<0.001	1.170 (1.028–1.331)	0.018

Supplementary Table S4. Univariate and Multivariable Cox Regression Analysis for PLR as a continuous variable.

**Variable**	**HR (95% CI)**	**p value**	**aHR (95% CI)**	**p value**
PLR	1.002 (1.001–1.002)	<0.001	1.001 (1.001–1.002)	<0.001
Heart Failure	3.822 (2.111–6.922)	<0.001	2.499 (1.210–5.161)	0.013
Gender	0.927 (0.670–1.284)	0.649	1.224 (0.816–1.837)	0.329
Arterial Hypertension	0.969 (0.716–1.311)	0.838	0.880 (0.604–1.282)	0.507
Diabetes Mellitus	1.629 (1.192–2.227)	0.002	1.364 (0.927–2.007)	0.115
Dyslipidemia	0.845 (0.568–1.259)	0.408	0.822 (0.524–1.291)	0.395
Chronic Kidney Failure	3.095 (2.040–4.695)	<0.001	1.502 (0.802–2.811)	0.204
Atrial Fibrillation	1.718 (1.118–2.641)	0.014	1.073 (0.609–1.890)	0.806
Anticoagulant use	1.565 (1.124–2.179)	0.008	1.201 (0.785–1.837)	0.399
Diuretic use	2.058 (1.493–2.835)	<0.001	1.634 (1.110–2.405)	0.013
B-blockers use	1.117 (0.819–1.524)	0.484	0.759 (0.516–1.115)	0.160
Age	1.031 (1.018–1.044)	<0.001	1.012 (0.997–1.028)	0.125
tnt_HS	1.001 (1.001–1.003)	0.004	1.001 (1.001–1.003)	0.686
Serum creatinine	1.202 (1.104–1.308)	<0.001	1.149 (1.010–1.308)	0.035
